# Supplementary material for: Structure of orthoreovirus RNA chaperone σNS, a component of viral replication factories
Source: Nat Commun. 2024 Mar 19;15:2460. doi: 10.1038/s41467-024-46627-8 (PMC10950856; doi:10.1038/s41467-024-46627-8)
Supplement: Supplementary file 1 — Supplementary Information [file 41467_2024_46627_MOESM1_ESM.pdf]

Supplementary Information

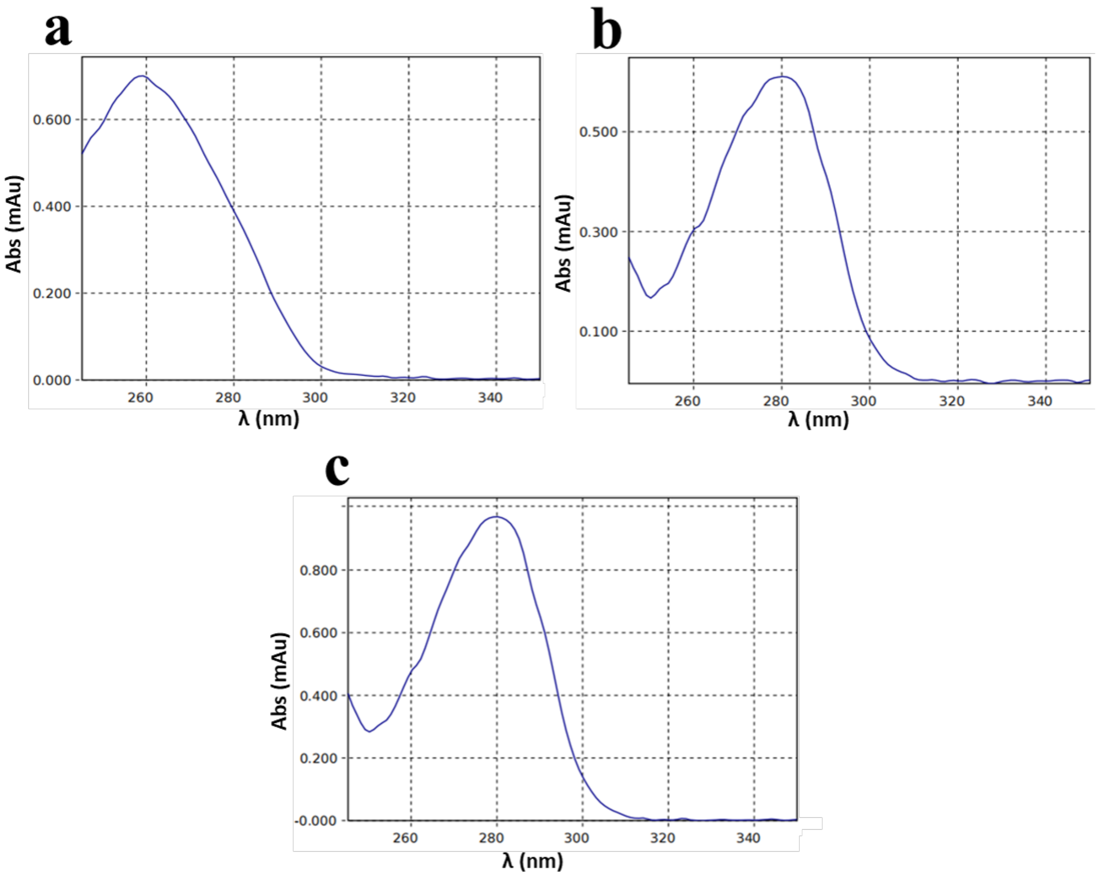

Fig. S1 Absorption spectrum from 240 to 340 λ (nm) of purified WT σNS and σNS-R6A. a This tracing corresponds to peak 1 in Fig. 1a for WT σNS, showing an absorbance maximum at 260 nm, indicating the presence of RNA along with protein. **b** This tracing corresponds to peak 2 in Fig. 1a for WT σNS, showing an absorbance maximum at 280 nm, indicating the presence of protein without RNA. **c** This tracing corresponds to the peak in Fig. 1a for σNS-R6A, showing an absorbance maximum at 280 nm, indicating the presence of protein without RNA. Absorption spectra were obtained using a biospectrometer (Eppendorf).

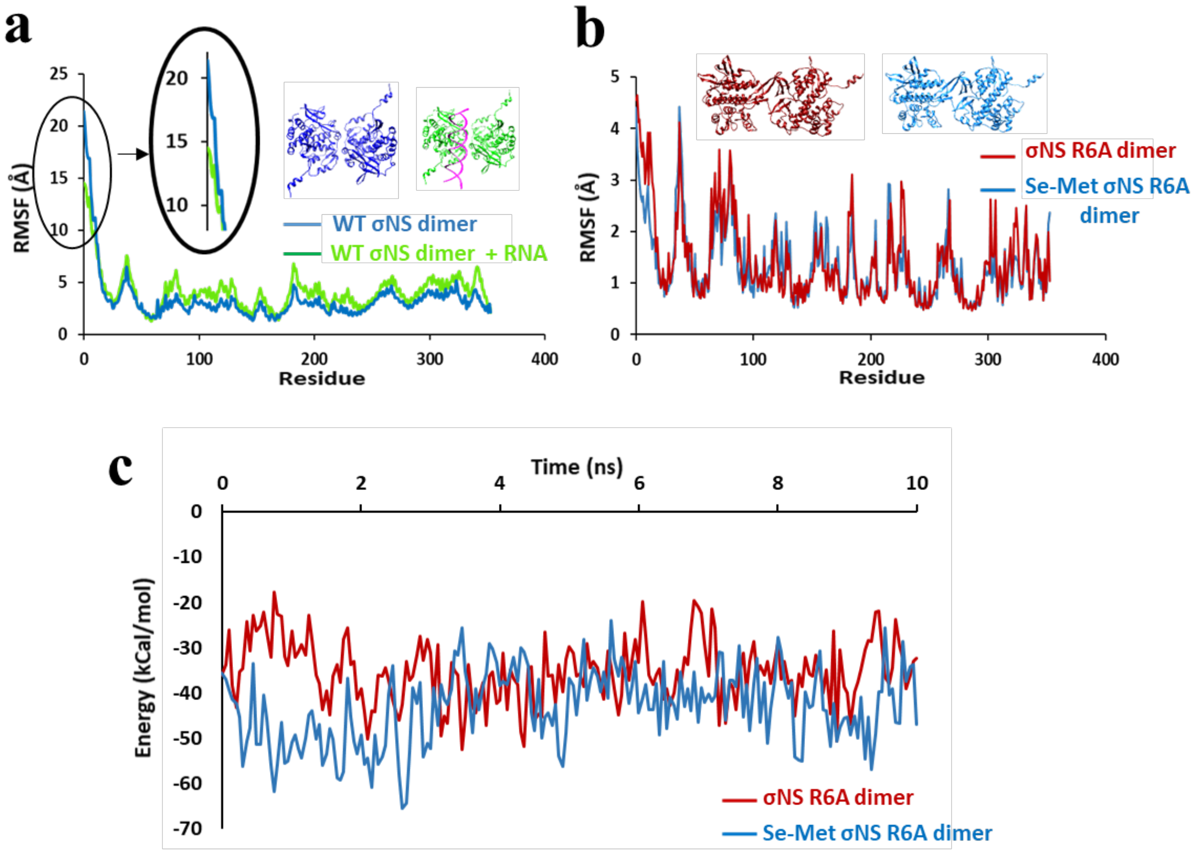

Fig. S2 MD simulation studies of WT σNS, σNS-R6A, and Se-Met σNS-R6A dimers. a The RMSF graph of the WT σNS dimer (blue) alone and in complex with a modeled double-stranded RNA (green) molecule after 10 nanoseconds of molecular dynamics simulation (MDS). **b** The RMSF graph of the σNS-R6A (red) and Se-Met σNS-R6A (blue) dimers linked by domain swapping N-terminal arms after 10 nanoseconds of MDS. **c** Interaction energy calculations of the σNS-R6A and Se-Met σNS-R6A dimers shown in **b**. The data for all the graphs are provided in the "Source Data" file.

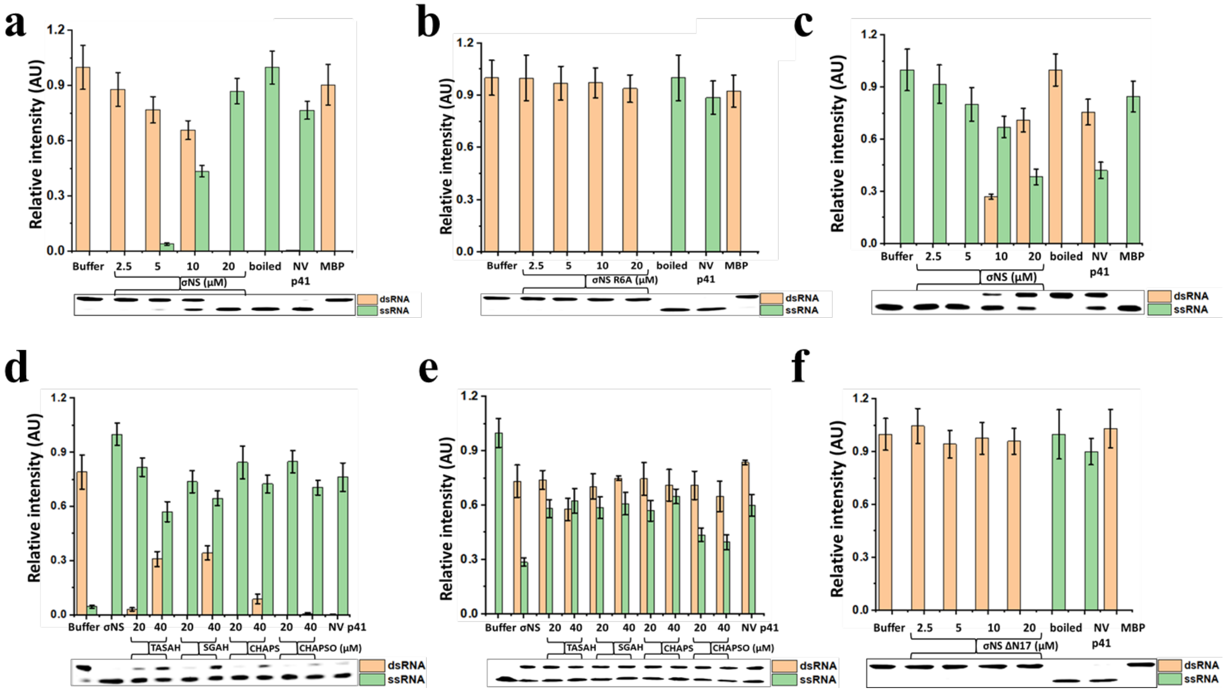

Fig. S3 RNA chaperone activity of σNS. Bar graphs and gel electrophoresis showing RNA helix-destabilizing and RNA-annealing activities of σNS. The dsRNA and ssRNA species are indicated by orange and green bars, respectively. **a** Helix-destabilizing activity of σNS. Increasing concentrations of WT σNS yield an increase in ssRNA and a decrease in dsRNA. **b** Helix-destabilizing activity of σNS R6A. **c** RNA strand-annealing activity of σNS. **d** Effects of bile acid derivatives on RNA helix-destabilizing and **e** RNA strand-annealing activities of σNS. **f** Helix-destabilizing activity of σNS ΔN17. The data for all the graphs, along with uncropped gels, are provided in the "Source Data" file.

Table S1. Oligonucleotides used in this study

**Oligonucleotide**	**Sequence (5' to 3')***
RNA1	CAUUAUCGGAUAGUGGAACCUAGCUUCGACUAUCGGAUAAUC
RNA2	AAUAAAGAUUAUCCGAUAGUCGAAGCUAGGUUCCACUAUCCGAUAAUGAAAUAA
RNA3	GAUUAUCCGAUAGUCGAAGCUAGGUUCCACUAUCCGAUAAUG

* HEX-labeled strand is in boldface.

Table S2. RMSF values of the N-terminal arms of the WT σNS dimer alone and in complex with RNA

Amino acid	RMSF WT σNS dimer	RMSF WT σNS dimer + RNA
1	21.40	14.53
2	20.04	14.36
3	18.17	13.91
4	17.22	12.76
5	16.85	12.26
6	16.87	13.15
7	14.00	11.29
8	12.58	9.95
9	10.99	9.47
10	10.91	9.71
11	11.16	9.94
12	8.86	8.15
13	8.55	7.28
14	6.84	7.29
15	5.61	5.85
16	5.27	6.16
17	4.34	5.00
